# Removals

**Published:** 1896-06

**Authors:** 


### Removals.

DR. L. J. MCADAM, of Buffalo, announces that he has changed his residence to 341 Jersey street, corner of Normal avenue, and that he will retain his present office at 108 Austin street, in which Dr. C. M. Brown will be associated. Hours: 341 Jersey street, until 9. A. M., 1 to 3 P. M.; at 108 Austin street, 10 A. M., 7 to 8 P. M.; Sunday, 4 to 6 P. M.

---

DR. LORENZO BURROWS, of Albion, N. Y., has moved to 388 Franklin street, Buffalo, where he will continue the practice of ophthalmology and otology.

---

DR. JOHN E. BACON, of Buffalo, announces the removal of his office and residence from 149 Franklin street to 79 Niagara Square. Hours: 10 to 12 A. M., 2 to 4 P. M., 7 to 8 P. M.

Dr. Jane N. Frear announces a change of location from 145 Seventh street to 28 Orton place, Buffalo. Hours: 8 to 10 a. m., 1 to 3 p. m. and 7 p. m.

---

Dr. Frederick Preiss and Dr. William Preiss have removed from 115 Franklin street to 160 Franklin street, Buffalo, N. Y.

---

Dr. J. J. Finerty, of Buffalo, has removed from 220 Franklin street to 266 Franklin street.

---

Dr. J. W. Putnam, of Buffalo, has removed from 388 Franklin street to 525 Delaware avenue.

---

Dr. F. H. Mills, of Buffalo, has removed from 160 Franklin street to — Huron street.

---

Dr. John H. Pryor has removed from 253 Allen street, Buffalo, to 56 Allen street.

